# How do women comply with cancer screenings? A study in four regions of France

**DOI:** 10.1186/s12905-023-02311-5

**Published:** 2023-04-21

**Authors:** Nathalie Duchange, Marie Poiseuil, Quentin Rollet, Christine Piette, Mathilde Cosson, Marie-Christine Quertier, Grégoire Moutel, Sylviane Darquy

**Affiliations:** 1 Normandie univ, UNICAEN, Inserm U1086, ANTICIPE, Caen, 14000 France; 2 Cancer et expositions environnementales, Univ. Bordeaux, Inserm U1219, EPICENE, Bordeaux, 33000 France; 3 Centre Régional de Coordination du Dépistage des Cancers (CRCDC), Bretagne, France; 4 Centre Régional de Coordination du Dépistage des Cancers (CRCDC) Normandie, Paris, France

**Keywords:** Organised cancer screening, Women, Breast cancer, Colorectal cancer, Cervical cancer, Questionnaire

## Abstract

**Background:**

This article looks at the behaviour of women facing different cancer screening options available to them from the age of 50 onward. The study was conducted in 2019 in four departments of the French territory with the objective of identifying the factors that influence acceptance of a population-based screening proposal.

**Methods:**

A questionnaire was sent to women who had received three invitations to organised screenings (OS) for both breast and colorectal cancer. The categories of participants in both OS were designed from data from the regional cancer screening coordination centres in each department. Participation in opportunistic cervical cancer screening was evaluated as self-reported data.

**Results:**

4,634 questionnaires were returned out of the 17,194 sent, giving a global return rate of 27%. The highest rate of return (73.5%) was obtained from women who had participated at least once in both breast and colorectal cancer OS. An intermediate rate was obtained from women participating in breast cancer OS only (18.7%). Poor levels of return came from women who had participated in colorectal cancer OS only (3.6%) and from non-participants (4.1%). Our results suggest that women with lower educational levels tend to be the most regular attendants at OS (50.3%), compared to highly educated women (39.7%). 11.8% of women were overdue in their opportunistic cervical cancer screening. This percentage rose to 35.4% in the category of non-participants. In addition, women’s comments provide a better understanding of the reasons for irregular attendance and non-participation.

**Conclusion:**

Overall, similar behaviours towards screening were observed in the four departments. Our analysis suggests that participation in one cancer OS increases the likelihood of participating in others. This adhesion could be an interesting lever for raising women’s awareness of other cancer screenings.

**Supplementary Information:**

The online version contains supplementary material available at 10.1186/s12905-023-02311-5.

## Background

Three cancer screenings are promoted in France today and are integrated into national public health programmes. Organised screenings (OS) for breast and colorectal cancer were generalized in 2004 and 2009 respectively. Between 50 and 74 years of age, the target population receives a personal letter of invitation at home to perform a free mammography or to get a hemoccult test. Individuals must then retrieve the kit from their physician. Invitations are sent every two years by regional cancer screening coordination centres. Until 2018, cervical cancer screening was carried out on an individual basis -opportunistic screening - by health professionals and through pap smears. In accordance with the third French Cancer Plan (2014–2019), the gradual implementation of nationwide cervical cancer screening started in 2018 [1].

For the years 2018–2019, the national female OS participation rate was 49.3% for breast cancer [2] and 31.9% for colorectal cancer [3]. Screening for colorectal cancer is done using a test that detects the presence of occult blood in the stool. Since 2015, a new test has been introduced, the immunological test, which involves taking only one stool sample instead of three previously [4]. Although the latter is simpler to use, no significant impact on participation was found as measured in 2018–2019 [3]. Participation in cancer OS is on a voluntary basis and according to the principle of autonomy. This presumes that the target population is properly informed and fully autonomous in its decision-making. On the other hand, the aim of a public health system is to ensure that as many people as possible participate in it and to reduce social inequalities. Insufficient participation may challenge the efficiency of a public health programme. The whole question thus revolves around the relation between individual and collective stakes that conditions adhesion to a population-based screening proposal [5] and the information to be provided to allow autonomous and informed decision-making [6].

Studies on the barriers to, and the levers of participation in cancer screening have often considered each programme separately [7, 8]. However, as the cancer screening offer has expanded, recent studies have sought to understand the behaviour of the target populations concerned by different screenings as well as the regularity of participation in different screening rounds [9–12].

This study was based on a questionnaire sent to a large population of women concerned by breast, colorectal and cervical cancer screenings. The aim was to better understand how they behave towards each of the cancer screening invitations. We explored their participation in joint breast and colorectal cancer OS as well as in opportunistic cervical cancer screening. The reasons for irregular participation or non-participation were investigated. This paper presents the protocol, the questionnaire and the evaluation of the number of women in each participation profile.

## Methods

### Study

This is a descriptive study addressing women’ behaviour toward three cancer screenings through a questionnaire allowing both a quantitative and a qualitative approach. Four departments were selected in order to have a representative sample of urban, peri-urban and rural populations and to target a sufficient number of women for the methodology used.

### Population

Women aged 56 as of 1st January 2019 were the target of the study population in four territorial departments, each managed by a regional cancer screening coordination centre (CRCDC):” Gironde”, “Calvados”, “Orne” and “Ille et Vilaine”. As part of their public health mission, the CRCDC generate invitations and follow up with the persons concerned by the screening as desribed by Poiseuil et al. [13]. These data are transmitted by the National Health Insurance (Caisse Nationale de l’Assurance Maladie). The CRCDC are data controllers according to the EU General Data Protection Regulation 2016/679 (GDPR) [14, 15].

At this time, they thus received three sequential invitation letters two years apart for breast and cancer OS (at ages 50, 52 and 54). Women who expressed an opt-out from participating and women with a positive result (breast or colorectal) from the last screening test were not included.

### Categories

Based on the 6 invitations received, 4 categories were determined from the CRCDC database. The “Breast + Colorectal” category includes women who participated in both OS (at least once in each). The “Breast only” category includes women having participated in breast cancer OS only (at least once). The “Colorectal only” category includes women having participated in colorectal cancer OS only (at least once). The “No participation” category includes women having responded to none of the 6 invitations.

### Questionnaire structure

A questionnaire was sent to women according to the participation categories defined above. The questionnaire was composed of 5 sections covering questions about (1) the person, (2) participation in breast cancer OS, (3) participation in colorectal cancer OS, (4) participation in opportunistic cervical cancer screening and (5) the recipient’s vision of public health.

Sections 1, 4 and 5 were common to all categories. Sections 2 and 3 differed according to participation or not, as some questions were unsuitable. Finally, some specific questions were addressed to the non-participant category. The questionnaire consisted of 45 to 58 closed-ended questions. The opportunity to add open-ended comments was available for the majority of questions. The most complete questionnaire composed of 58 questions is supplied [see Additional file 1].

### Questionnaire mailing and return

The questionnaires were mailed from January 2019 to March 2019 by the four regional cancer screening coordination centres in charge of managing the OS at a local level. The mailing also included a letter of information describing the objectives and the bodies in charge of the project as well as a pre-stamped envelope for the return of the questionnaire. It also provided the address of a web site delivering information about the research teams, their previous work on cancer screening and news about the progress of the research. Vulgarized research results will also be available there. The questionnaires were returned anonymously. The returns ended in July 2019.

### Questionnaire entry and analysis

For each department, the returned questionnaires were entered into an Excel file according to a common grid. Quantitative analysis was performed in Excel. Our study has enabled us to build up a substantial database for future statistical analysis.

Description of the responding population by department and by categories in term of number and percentage. Qualitative answers were transcribed and manually analysed. Comments were categorized into themes before main themes and allocation of comments were decided upon. This to describe the reason for no participation the 3 breast and 3 cancer OS invitations, and the reason of participation to cervical cancer screening.

## Results

### Number of women in categories according to the CRCDC databases

Our study population of 17,194 women was determined from the data of the four regional screening cancer coordination centres as well as their repartition in the designed categories. As shown in Tables 1, 41.2% of women participated in both cancer OS (ranging from 35.2% in “Gironde” to 51.3% in “ Ille et Vilaine”); 29.8% participated in breast cancer OS only (from 24.3% in “Ille et Vilaine” to 34.1% in “Gironde”). Participation in colorectal cancer OS alone was infrequent, 4.2% in total with a similar range across the four structures. Finally, 24.8% did not participate in either program (from 20.1% in “Ille et Vilaine” to 27.2% in “Gironde”).

**Table 1:** Number of women in the designed categories: data from regional cancer coordination centres

*Category*	Gironde N = 7,240	Orne N = 2,176	Calvados N = 3,751	Ille et Vilaine N = 4,027	Total *N = 17,194
	n (%)	n (%)	n (%)	n (%)	n (%)
Breast + Colorectal (at least once for each)	2,550 (35.2)	921 (42.3)	1,544 (41.2)	2,067 (51.3)	7,082 (41.2)
Breast only (at least once)	2,470 (34.1)	551 (25.3)	1,127 (30.0)	977 (24.3)	5,125 (29.8)
Colorectal only (at least once)	254 (3.5)	141 (6.5)	149 (4.0)	175 (4.3)	719 (4.2)
No participation	1,966 (27.2)	563 (25.9)	931 (24.8)	808 (20.1)	4,268 (24.8)

*N corresponds to the total number of questionnaires sent

**Table 2A:** Number of questionnaires returned by department and category of participation to breast and colorectal cancer OS

*Category*	Gironde	Orne	Calvados	Ille et Vilaine	Total return by category
	n (%)	n (%)	n (%)	n (%)	n (%)
Breast + Colorectal (at least once for each)	1,223 (71.6)	414 (67.9)	737 (69.6))	1,034 (82.4))	3,408 (73.5)
Breast only (at least once)	360 (21.1)	126 (20.6)	222 (21.0)	160 (12.7)	868 (18.7)
Colorectal only (at least once)	60 (3.5)	33 (5.4)	36 (3.4)	37 (2.9)	166 (3.6)
No participation	66 (3.9)	38 (6.2)	64 (6.0)	24 (1.9)	192 (4.1)
Total return by department	1,709	611	1,059	1,255	4,634

### Description of the responding population

#### Questionnaire rate of return by department and category

4,634 questionnaires were returned yielding a global response rate of 27% (23.6% % in “Gironde”, 28.1% in “Orne”, 28.2% in “Calvados” and 31.2% in” Ille et Vilaine”). As shown in Table 2A, the rate of return by category was similar in the four regions with the highest level of return in the “Breast + Colorectal” category (ranging from 69.6 to 82.4%). The rate of return for women participating in one OS only was 18.7% (ranging from 12.7 to 21.1%) for breast cancer and 3.6% (ranging from 2.9 to 5.4%) for colorectal cancer. The “No participation” category showed a low rate of return, ranging from 1.9 to 6.2%.

We distinguished women who responded to all 6 invitations (full attenders) from those who responded only to some invitations (irregular attenders) (Table 2B). Full attenders represented 60.1% of the category and irregular attenders 39.9%. In the four departments, full attenders were those who answered the questionnaire the most.

**Table 2B:** Repartition of full and irregular attenders in the “Breast + Colorectal’ category

*“Breast + Colorectal”* *category*	Gironde n = 1,223	Orne n = 414	Calvados n = 737	Ille et Vilaine n = 1,034	Total n = 3,408
	n (%)	n (%)	n (%)	n (%)	n (%)
Full attenders (3 for each)	677 (55.4)	275 (66.4)	433 (58.8)	664 (64.2)	2,049 (60.1)
Irregular attenders (at least once for each)	546 (44.6)	139 (33.6)	304 (41.2)	370 (35.8)	1,359 (39.9)

#### Level of education according to categories

Table 3 shows that 43% (n = 1,981) of total respondents had a level lower than the French baccalaureate (high school diploma) and 56% (n = 2,583) had the baccalaureate or a level above (no answer n = 70). The percentages of women according to level of education in each category were comparable across the four regions. Only a difference was observed among the full attenders, where the number of women with an education level below the baccalaureate was higher (50.3%) than those with the baccalaureate (39.7%), in the four regions together.

**Table 3:** Breakdown in categories according to level of education in the four departments

Level of education	Gironde		Orne		Calvados		Ille et Vilaine		Total	
	p = 0.001		p = 0.123		p = 0.0002		p = 0.0007		p < 0.001	
	<BAC	> = BAC	<BAC	> = BAC	<BAC	> = BAC	<BAC	> = BAC	<BAC	> = BAC
	n = 671	n = 1,002	n = 343	n = 264	n = 479	n = 566	n = 488	n = 751	n = 1,981	n = 2,583
	n (%)	n (%)	n (%)	n (%)	n (%)	n (%)	n (%)	n (%)	n (%)	n (%)
*“Breast + Colorectal”*										
Full attenders	308 (45.9)	356 (35.5)	168 (49.0)	104 (39.4)	225 (47.0)	203 (35.9)	295 (60.5)	362 (48.2)	996 (50.3)	1025 (39.7)
Irregular attenders	190 (28.3)	344 (34.3)	70 (20.4)	68 (25.8)	126 (26.3)	173 (30.6)	115 (23.6)	250 (33.3)	501 (25.3)	835 (32.3)
Breast only	129 (19.2)	221 (22.1)	69 (20.1)	57 (21.6)	77 (16.1)	142 (25.1)	57 (11.7)	101 (13.4)	332 (16.8)	521 (20.2)
Colorectal only	20 (3.0)	39 (3.9)	19 (5.5)	14 (5.3)	16 (3.3)	20 (3.5)	14 (2.9)	23 (3.1)	69 (3.5)	96 (3.7)
No participation	24 (3.6)	42 (4.2)	17 (5.0)	21 (8.0)	35 (7.3)	28 (4.9)	7 (1.4)	15 (2.0)	83 (4.2)	106 (4.1)

### Analysis of the answers

#### Reasons for non-response to the three breast and three colorectal cancer OS invitations

Table 4A presents thematic comments from women as regard to breast cancer OS. A few quotes were selected to illustrate the themes. Similar comments were found in the four departments. For those who participated 1 or 2 times, omission, negligence, procrastination and lack of time were the major reasons reported (36.8%). Negligence was the most frequently used term. In 23.3% of comments, the women said that they had had mammogram(s) outside of OS. Either they had had one mammogram shortly before being invited in the OS at the age of 50 or they had had prescription from their gynaecologist before he/she retired. There were also occasional alerts: “*I had to have a mammogram between 2 invitations”.* Some women did not distinguish between follow-ups by their physician and OS: *“I do it every two years, either with my gynaecologist or with you, I don’t know*”. Mammograms outside of OS for medical reasons - family history, follow-up of cysts or other medical problems- represented 10.1% of the comments. Those helped to clarify why respondents alternated between being in and out of OS: “*Following a suspicion in 2012, I was regularly monitored at 6 months, then 1 year before entering the OS*”. Finally, practical considerations and other priorities were two items which helped to understand why some women were irregular attendants.

**Table 4:** Reasons for non-participation to all 3 breast cancer OS (4 A) and all 3 colorectal cancer OS (4B)

4-A	Breast OS participation		
*Thematic comments*	1–2 N = 437	0 N = 146	Total N = 583
	n (%)	n (%)	n (%)
Omission/negligence/procrastination/no time	**161 (36.8)**	19 (13.0)	180 (30.9)
Mammography outside OS with the physician (opportunistic)	**102 (23.3)**	**38 (26.0)**	140 (24.0)
Mammography outside OS for medical reasons including breast cancer history	44 (10.1)	**54 (37.0)**	98 (16.8)
Reluctance to screening (pain, doubts, toxicity of radiation, too frequent)	37 (8.5)	11 (7.5)	48 (8.2)
Practical considerations (appointment complicated, distance, not remembering receiving invitation)	35 (8.0)	7 (4.8)	42 (7.2)
Not motivated/not concerned	20 (4.6)	7 (4.8)	27 (4.6)
Other priority	23 (5.3)	1 (0.7)	24 (4.1)
Fear of result	8 (1.8)	5 (3.4)	13 (2.2)
Previous bad experience	7 (1.6)	4 (2.7)	11 (1.9)
**4-B**	**Colorectal OS participation**		
*Thematic comments*	1–2 N = 823	0 N = 597	Total N = 1,420
	n (%)	n (%)	n (%)
Omission/negligence/procrastination/no time	**228 (27.7)**	115 (19.3)	343 (24.2)
Colonoscopy for medical reasons including colorectal cancer history	83 (10.1)	**125 (20.9)**	208 (14.6)
Not motivated/not concerned	96 (11.7)	103 (17.3)	199 (14.0)
Test complicated/constraining	76 (9.2)	86 (14.4)	162 (11.4)
Difficulty of access to the test	109 (13.2)	32 (5.4)	141 (9.9)
Unpleasant examination, disgust	38 (4.6)	49 (8.2)	87 (6.1)
Fear of result/ Fear of coloscopy in case of positive result	24 (2.9)	55 (9.2)	79 (5.6)
Difficulty with the first test (FOBT)	62 (7.5)	6 (1.0)	68 (4.8)
Not remembering having received invitation(s)	60 (7.3)	5 (0.8)	65 (4.6)
Reluctance to screening (no confidence in reliability, too frequent, too much screening)	25 (3.0)	9 (1.5)	34 (2.4)
Other priority	22 (2.7)	12 (2.0)	34 (2.4)

146 comments came from non-participants. 63% said that they had a regular follow-up outside of OS, 37% for medical reasons and 26% with their physician: “*I combine this screening with my gynaecological appointment*”. Reluctance about screening, lack of motivation/a feeling of not being concerned were items that were similar in terms of percentages to comments from those of the 1–2 participants. These women evoked their Healthy lifestyles and no cancer history. Some women were reluctant because the exam is too painful: “I’m scared, getting my breast crushed makes me faint”; “I call them torture devices, they hurt a lot”. Fear of over-diagnosis and over-treatment were rarely mentioned.

Table 4B presents comments from women who participated 1 or 2 times in colorectal cancer OS. Omission, negligence, procrastination and lack of time were also the first reasons reported for non-response to all invitations (27.7%). Comments were often expressed in the past sense, pointing to a gradual take-up: “*I didn’t think it was that important. Now I am convinced*”. Some encountered difficulties with the use and access to the test: “*Difficult to use the first time, so I had given up*”.

“*I have not yet participated in the third invitation because I have to go to my practitioner to pick up the test*”. Some of the women received the test at home over a period as part of a pilot intervention. For those, incomprehension was even greater: “*As the screening kit is no longer supplied with the mail and I don’t go to the doctor, I left it on hold*”. “*I am waiting to receive the test at home*”. Finally, 9.2% still found the test complicated: “*I found it constraining although I know that now the procedure has been simplified*”. And the test was also found unpleasant: “*I found it degrading to have my stool analysed*”.

Comments from non-participants show that the use of colonoscopies was high (20.0%). More women did not feel concerned (17.3%) or they commented on their lack of awareness: “*No knowledge of this cancer*”, “*I am less aware of colorectal cancer, perhaps because there is no colorectal cancer among my family and friends*”. Some used the present tense to report that they did not wish to participate in this screening: “*For the moment I am undecided*”, “*I don’t think I’m at risk*”. When it came to prioritising, breast cancer screening took precedence: “*Took this examination at the second level compared to the mammogram*”. 14.4% presumed that the test was complicated or have tried but have given up: “*Complicated procedure, I think*”, “*I did not know how to use the equipment*”. Fear and disgust were also factors more frequently mentioned by those who had never performed the test compared to those who had done so: fear (9.2% versus 2.9%) and disgust (8.2% versus 4.6%). Reluctance about screening represented 2% of the comments. This was associated with the stress induced by medical exams or with their number: “*Fed up with screenings*”, “*I do not wish to be kept under stress due to intensive screening*”, “*Feeling that you spend your time doing exams*”.

#### Opportunistic participation in cervical cancer screening

Opportunistic screening was evaluated in the questionnaires (Table 5). Table 5A shows that a high percentage of respondents had had a pap smear in the last 3 years. Women who had had a hysterectomy were not counted. Those never screened represented 2.4% and the number of overdue women ranged from 10.6 to 12.5% in the four departments. Table 5B shows the distribution across categories. The ‘No participation’ category shows a largely higher percentage (34.4%) of those never screened or who were overdue in comparison to the other categories.

**Table 5:** Participation to cervical cancer screening (opportunistic) according to the departments (5 A) and categories (5B)

5-A				
*Department*	Pap test ≤ 3 years	Pap test >3 years (overdue)	Never-screened	Hysterectomy No answer
	n (%)	n (%)	n (%)	n (%)
Gironde (N = 1,709)	1,381 (80.8)	205 (12.0)	18 (1.1)	105 (6.1)
Orne (N = 611)	479 (78.4)	65 (10.6)	18 (2.9)	49 (7.5)
Calvados (N = 1,059)	832 (78.6)	133 (12.5))	44 (4.2)	50 (4.7)
IlLe et Vilaine (N = 1,255)	1,028 (81.9))	142 (11.3)	29 (2.3)	56 (4.4)
Total (N = 4,634)	3,720 (80.3)	545 (11.8)	109 (2.4)	260 (5.6)
**5-B**				
*Category*	Pap test ≤ 3 years	Pap test >3 years (overdue)	Never-screened	Hysterectomy No answer
	n (%)	n (%)	n (%)	n (%)
Breast + Colorectal (N = 3,408)	2,824 (82.9)	334 (9.8)	58 (1.7)	192 (4.1)
Breast only (N = 868)	670 (77.2)	128 (14.7)	30 (3.5)	40 (4.6)
Colorectal only (N = 166)	123 (74.1)	17 (7.2)	8 (4.8)	18 (10.8)
No participation (N = 192)	103 (53.6)	**66 (34.4)**	13 (6.8)	10 (5.2)
Total (N = 4,634)	3,720 (80.3)	545 (11.8)	109 (2.4)	260 (5.6)

## Discussion

Among the 17,194 women in our study population, 41.2% participated at least once in both breast and colorectal OS. Around one third participated in breast cancer OS only while participation in colorectal cancer OS alone was very infrequent. Non-participants represent a quarter of the study population. The distribution across categories was quite similar in the four departments. This reflects the higher participation in breast cancer OS than in colorectal cancer OS in France, as well as the high number of non-participants [2, 3, 16]. It may be explained in part by the fact that the colorectal cancer OS was implemented later, and that fewer awareness campaigns were conducted.

The global return rate was 27%, i.e., 4,634 analysable questionnaires out of the 17,194 sent. This rate was homogenous across the departments. However, it differed according to the category. The highest rate was observed for participants in both OS types, especially for the full attenders. Thus, adhesion to OS was correlated with the return rate. Participants in a single OS had an intermediate response rate and non-participants responded little. The difficulty in reaching non-participants was not unexpected, as they are also often unwilling to discuss the matter [17].

The distribution of respondents’ level of education shows that a majority felt concerned by the questionnaire. The distribution was comparable in each category in the four regions. Interestingly, among the full attenders, the majority of women had the lowest level of education in all four departments. Thus, in our study, the level of education does not seem to represent a barrier to OS. This is in line with other studies [18, 19] which have shown that while opportunistic screening is associated with a higher educational level, educational inequalities are lower in European countries when organised screening is in place, independently of the cancer type. In France, Kelly et al. [20] also suggested that organised screening for breast and colorectal cancer has the potential to reduce socioeconomic inequalities.

Irregular attenders at breast cancer OS most often reported neglect, forgetfulness or procrastination. Although the invitation letter was welcomed as a reminder, the 2-year interval sometimes appeared to be difficult to adhere to. Some women commented that they had missed the first appointment because of a recent mammogram before the age of 50, or that they had attended the OS when their gynaecologist had retired. Entry into the OS system is key, as several studies have shown that first attenders have a high probability of re attending [21–23]. On the other hand, many non-participants declared that they had had opportunistic mammograms. This is a well-established fact in the French context [24, 25]. It has been shown that women from disadvantaged areas tend to participate in OS, whereas women who do opportunistic mammograms argue that they can and want to be responsible for their own health, to have the “freedom to manage” [26]. In the wake of the reform of the French national breast cancer programme, one recommendation from an expert committee was to limit the use of opportunistic screening in the absence of medical reasons after the age of 50 [27]. This is in order to prioritise OS that ensure equal quality throughout the territory, traceability and the efficiency of a population-based screening.

Absence of motivation or reluctance towards breast cancer screening due to the fear of over diagnosis was rarely mentioned. Few women said that they did not want to be screened. Those who did either pointed to their healthy lifestyle or rejected mammographs because of pain or fear of radiation. This tends to show that women attending screening, whether in or out of OS, have put these barriers to one side. Indeed, it has been shown that information about the benefits and harms of breast cancer screening help women make an informed decision but do not affect participation [28–30].

Irregular attenders at colorectal cancer OS also mentioned neglect, forgetfulness or procrastination. Difficulty of access to the test was a concern, especially for those who rarely consult their physician. They do not understand why they cannot simply get the test at a pharmacy or why it is not sent to their home. The incomprehension is even greater among women who received the test at home in a pilot intervention. They were just expecting to receive the test again at home. Finally, a number of comments pointed to the difficulty of the test, which led them to give up. However, many acknowledged the fact that the immune test was much simpler.

A significant number of non-participants in colorectal cancer OSreported having colonoscopies for medical reasons, including family history. Such reasons are expected to be reported to the regional cancer screening coordination centres. However, this seems not always to be the case. Interestingly, the test appears to be more complicated for those who have never done it than for those who have. The same applies to disgust or the unpleasantness of the test. The percentage of women who do not feel concerned by colorectal screening is higher than for breast cancer screening. A lack of awareness of this cancer is evident in the comments. Again, first-round participation appears to be a strong indicator of participation in future rounds, prompting the emphasis on initial participation. Barriers to and levers for entering the programme have been documented [31–33], and Mandrik et al. [8] reviewed the literature on policy procedures with a positive impact on participation among women invited to the first round of breast cancer OS.

Only a small percentage of our respondents said that they had never had opportunistic cervical cancer screening. Overdue respondents (test not done in the last 3 years) are more common. The women who did not participate in cancer OS were those with the highest percentages of overdue tests. In France, the screening coverage rate for cervical cancer in women aged 25–65 was estimated at 52% for the period 2012–2014, falling to 43.9% in the 55–59 age group [34]. This corresponds to one pap smear per woman over the recommended 3-year interval.

A major limitation of this study is the low response rate of women who do not participate in organised screening, representing a major selection bias. Furthermore, as the study was carried out on a voluntary basis and in 4 departments, the rates observed in this study are not representative of participation at national level. A declarative bias may also have been generated in the women’s responses. However, the study found a clear difference between awareness of breast cancer screening and that of colorectal cancer screening, and the many comments made within open-ended questions offer a better understanding of the reasons for behaviours toward each screening.

## Conlusions

Our results suggest that participation in one cancer OS may increase the likelihood of participating in others. Since participation in breast cancer OS is higher than in colorectal cancer OS, this adhesion could be an interesting lever for raising women’s awareness about other cancer screenings. Our data will allow us to analyse in more detail the elements that condition or improve entry into a programme and the remaining in successive rounds. Finally, our results suggest that lower education levels may be associated with higher attendance in successive rounds of invitations. The women surveyed often commented that OS is an opportunity for them, thus acknowledging the role of OS in combatting health inequalities.

A better understanding of participation profilescould enable stakeholders to adapt their discourse and information modalities. Uniform collective information for all does not account for the singularity of behaviour, lifestyle or social level. For this reason, personalised support could be reinforced for screening. In this way, public health action could be more effective and could optimise the participation rate in screening, with the aim of both individual and collective benefits.

In terms of information, this implies that communicating on one screening may have an impact on the other type and that it is important to place the emphasis on initial invitations [8, 35, 36]. Information and support should help to enable entry into OS rather than relying on individual approaches. Health professionals are pivotal in guiding women towards this goal.

## Electronic supplementary material

Below is the link to the electronic supplementary material.

**Additional File 1**: Questionnaire sent to women aged 56 in four French departments

## Data Availability

The data are available from the corresponding author on reasonable request.

## Abbreviations

OS: Organised Screening
