# Supplementary material for: How do women comply with cancer screenings? A study in four regions of France
Source: BMC Womens Health. 2023 Apr 21;23:190. doi: 10.1186/s12905-023-02311-5 (PMC10122322; doi:10.1186/s12905-023-02311-5)
Supplement: Supplementary file 1 — Additional File 1: Questionnaire sent to women aged 56 in four French departments [file 12905_2023_2311_MOESM1_ESM.pdf]

A/ GENERAL QUESTIONS

For each question, select the box that corresponds to your situation ☒

1. What is your family situation?

☐ Married/Civil union/Couple ☐ Divorced/separated
☐ Single ☐ Widow

2. Do you have children? ☐ Yes ☐ No

3. What is your highest level of education?

☐ Primary education
☐ Secondary education without *brevet des collèges*
☐ Secondary education with *brevet des collèges*
☐ Baccalaureate
☐ Baccalaureate + 2 years
☐ Superior to Baccalaureate +2 years

4. What is (or was) your profession? ……………………………………………………………………………

5. What is your current situation?

☐ Professional activity
☐ Unemployed
☐ Retired
☐ Other, please specify ……………………………………………………………………………

6. What distance do you cover to go to your general practitioner? (one-way)

☐ 0 to 1 km
☐ 1 to 10 km
☐ 10 to 30 km
☐ More than 30 km
☐ I don't have a general practitioner **(go to question 9)**

7. If you have a general practitioner, how often do you consult him or her?

☐ More than once a year
☐ Once a year
☐ Very rarely, please specify ……………………………………………………………………

Name of the regional coordination cancer screening centre

Code category

8. How long does it take to get a consultation appointment?

☐ Less than 1 week

☐ Less than 1 month

☐ More than 1 month

9. What distance do you cover to go to your gynaecologist?

☐ 0 to 1 km

☐ 1 to 10 km

☐ 10 to 30 km

☐ More than 30 km

☐ I don't have a gynaecologist **(go to question 12)**

10. If you have a gynaecologist, how often do you consult him or her?

☐ At least once a year

☐ Less than once a year

☐ Very rarely, please specify: ………………………………………………………………

11. How long does it take to get a consultation appointment?

☐ Less than 1 week

☐ Less than 1 month

☐ More than 1 month

B/ PARTICIPATION IN SCREENING PROGRAMMES

Since the age of 50, you have been receiving letters inviting you to organised breast and colorectal cancer screenings

➢ ORGANISED BREAST CANCER SCREENING PROGRAMME

12. Did you participate in the organised breast cancer screening programme in response to the invitations you received at 50, 52 and 54 years of age?

Tick one box per line	Yes	No	Do not remember
1st invitation at 50	☐	☐	☐
2nd invitation at 52	☐	☐	☐
3rd invitation at 54	☐	☐	☐

If you answered yes to all 3 invitations <u>go directly to **question 14**</u>

13. If you did not answer yes to all 3 invitations, can you explain why?

………

………

Do you plan to participate in the future? ☐ Yes ☐ No ☐ Do not know

If yes, for what reason(s)? …………………………………………………………………………………………………

………

If no, for what reason(s)?……………………………………………………………………………………………………

………

14. Do you have mammograms outside the organised screening programme?

☐ Yes ☐ No ☐ Do not know

If yes, how often? ☐ Every year ☐ Every 2 years

☐ Every 3 years ☐ Above 3 years

If so, can you explain why you have mammograms outside the organised breast cancer screening?

………

………

15. Did you have mammograms before the age of 50?

☐ Yes ☐ No

If yes, for what reason(s)?

………

16. Invitation letters you received to organised breast cancer screening:

**Tick one box per line**	**Yes**	**No**	**No opinion**
Did they motivate you to participate?	☐	☐	☐
Did they appear intrusive to you?	☐	☐	☐
Would you prefer not to receive these invitations anymore?	☐	☐	☐
Did they provide you with information that you did not have?	☐	☐	☐
If so, can you explain? ……………………………………………………………………………… ………			
Did they cause you anxiety?	☐	☐	☐
If so, can you explain? ………………………………………………………………………………			

Name of the regional coordination cancer screening centre

Code category

17. Apart from these invitation letters, how did you mainly become aware of breast cancer screening? (one response only)

☐ Through a physician, which one? ○ General practitioner
○ Gynaecologist
○ Other: ………………………………

☐ Through prevention campaigns

☐ Through a case of breast cancer among the people I know

☐ I consider this to be part of the necessary medical follow-up

☐ Other, please specify: ……………………………………………………………………………………

18. Do you usually have the mammogram after receiving the invitation letter?

☐ Yes ☐ No, I am waiting for a second reminder letter

19. What do you think about breast cancer screening?

Tick one box per line	Yes	No	No opinion
I find it useful	☐	☐	☐
I feel concerned	☐	☐	☐
It is a chance to benefit from an organised national system	☐	☐	☐

If you wish, you can explain:

……

……

20. What are your reservations about breast cancer screening?

Tick one box per line	Yes	No	No opinion
I find the examination painful	☐	☐	☐
I am afraid of the result	☐	☐	☐
I am afraid I will have to pay	☐	☐	☐
I have other priorities	☐	☐	☐

Other(s): ……….

……

21. Do your relatives encourage you to participate in the breast cancer screening?

☐ Yes ☐ No

22. Do you yourself encourage your relatives to participate?

☐ Yes ☐ No

Name of the regional coordination cancer screening centre

Code category

➢ YOUR EXPERIENCE

23. How would you evaluate your overall experience with the breast cancer screening process?

☐ Good ☐ Moderate ☐ Bad ☐ No opinion

If you wish, you can explain:

………

………

24. For you, mammography screening is a process which is (Tick one box per line):

→ *On a practical level (making appointments, going to the centre, etc.)*

☐ Simple ☐ Complicated ☐ No opinion

→ *On a physical level*

☐ Neutral ☐ Unpleasant ☐ No opinion

→ *At the psychological level*

☐ Neutral ☐ Stressful ☐ No opinion

If you wish, you can explain:

………

………

25. Did you stop participating because of a negative experience(s) with cancer screening?

☐ Yes ☐ No

If you wish, you can explain:

………

………

26. Have you ever received a result showing a mammography abnormality that was not confirmed by further examinations (false positive)?

☐ Yes ☐ No ☐ Do not know

If so, how did you feel about it?

………

………

Did this ☐ Reinforce your motivation to participate in the next screening round
☐ Decrease your motivation to participate in the next screening round
☐ Have no effect

Name of the regional coordination cancer screening centre

Code category

❖ ORGANISED COLORECTAL CANCER SCREENING PROGRAMME

27. Did you participate in the organised colorectal cancer screening programme in response to the invitations you received at 50, 52 and 54 years of age?

Tick one box per line	Yes	No	Do not remember
1st invitation at 50	☐	☐	☐
2nd invitation at 52	☐	☐	☐
3rd invitation at 54	☐	☐	☐

If you answered **yes** to all 3 invitations go to question 29.

28. If you did not answer yes to all 3 invitations, can you explain why?

……

……

Do you plan to participate in the future? ☐ Yes ☐ No ☐ Do not know

If yes, for what reason(s)?

……

If no, for what reason(s)?

……

29. Do you have colonoscopies outside the organised screening programme?

☐ Yes ☐ No ☐ Do not know

If so, how frequently?

☐ Less than 4 years ☐ Every 4 years ☐ More than 4 years

30. Can you explain why you have colonoscopies **outside the organised programme**?

……

……

Name of the regional coordination cancer screening centre

Code category

31. Invitation letters you received to organised colorectal cancer screening:

Tick one box per line	Yes	No	No opinion
Did they motivate you to participate?	☐	☐	☐
Did they appear intrusive to you?	☐	☐	☐
Would you prefer not to receive these invitations anymore?	☐	☐	☐
Did they provide you with information that you did not have?	☐	☐	☐
If so, can you explain? ………………………………………………………………………………………… ………			
Did they cause you anxiety?	☐	☐	☐
If so, can you explain? ……………………………………………………………………………… ………			

32. Apart from these invitation letters, how did you mainly become aware of colorectal cancer screening? (one response only)

☐ Through a physician, which one? ○ General practitioner
○ Gastroenterologist
○ Other: ………………………………
☐ Through prevention campaigns
☐ Through a case of colorectal cancer among the people I know
☐ I consider this to be part of the necessary medical follow-up
☐ Other, specify: ………………………………………………………………………………

33. Do you usually have the mammogram after receiving the invitation letter?

☐ Yes ☐ No, I am waiting for a second reminder letter

34. What do you think about colorectal cancer screening?

Tick one box per line	Yes	No	No opinion
I find it useful	☐	☐	☐
I feel concerned	☐	☐	☐
It is a chance to benefit from an organised national system	☐	☐	☐

If you wish, you can explain: ………………………………………………………………………………………
………

Name of the regional coordination cancer screening centre

Code category

35. What are your reservations about colorectal cancer screening?

Tick one box per line	Yes	No
I find the test complicated to do at home	☐	☐
I am afraid of the result	☐	☐
I am afraid I will have to pay	☐	☐
I am afraid of a colonoscopy if I test positive	☐	☐
I have other priorities	☐	☐

Other(s): ………

36. Do your relatives encourage you to participate in the colorectal cancer screening?

☐ Yes ☐ No

37. Do you yourself encourage your relatives to participate?

☐ Yes ☐ No

➢ YOUR EXPERIENCE

38. How would you evaluate your overall experience with the colorectal cancer screening process?

☐ Good ☐ Moderate ☐ Bad ☐ No opinion

If you wish, you can explain:

……

……

39. For you, carrying out the colorectal test is a process which is (Tick one box per line):

→ *On a practical level (access to the test, realisation, sending the test, etc.)*

☐ Simple ☐ Complicated ☐ No opinion

→ *On a physical level*

☐ Neutral ☐ Unpleasant ☐ No opinion

→ *At the psychological level*

☐ Neutral ☐ Stressful ☐ No opinion

If you wish, you can explain:

……

……

Name of the regional coordination cancer screening centre

Code category

40. Did you stop participating because of negative experience(s)?

☐ Yes ☐ No

If you wish, you can explain:

………

41. Have you ever received a result showing an abnormal colorectal cancer screening test result that was not subsequently confirmed by colonoscopy (false positive)?

☐ Yes ☐ No ☐ Do not know

If so, how did you feel about it?

………

………

Did this
- ☐ Reinforce your motivation to participate in the next screening round
- ☐ Decrease your motivation to participate in the next screening round
- ☐ Have no effect

❖ CERVICAL CANCER SCREENING

Organised cervical cancer screening will be implemented in 2019. Currently, this screening is offered by health professionals as part of routine care.

42. Have you ever had a cervical screening test (Pap smear)?

☐ Yes ☐ No

43. At which age approximately did you start taking the test?

☐ Before 20 ☐ 20 to 30
☐ 30 to 40 ☐ 40 to 50
☐ After 50

44. At what age did you have your first sexual intercourse?

☐ Before 20 ☐ 20 to 30
☐ 30 to 40 ☐ 40 to 50
☐ After 50 ☐ I have never had sexual intercourse

45. Who is(are) the health professional(s) who performs the smear test?

☐ General practitioner ☐ Gynaecologist
☐ Midwife ☐ Other, specify: ………………………….

46. How frequently do you carry out the test?

☐ Once a year ☐ Every 2 years
☐ Every 3 years ☐ More than 3 years

47. When was the last time you had a smear?

☐ 1 year ago ☐ 2 years ago
☐ 3 years ago ☐ More than 3 years ago

48. You have been made aware of cervical cancer screening mainly (one response only):

☐ By a health professional:
- ○ General practitioner
- ○ Gynaecologist
- ○ Midwife
- ○ Other, specify: ……………………………

☐ Through prevention campaigns
☐ Through a case of cervical cancer among the people I know
☐ I think this is part of the gynaecological follow-up
☐ Other, specify: ……………………………………………………………………………………………..

49. What do you think about cervical cancer screening?

**Tick one box per line**	**Yes**	**No**	**No opinion**
I find it useful	☐	☐	☐
I feel concerned	☐	☐	☐
I am in favour of an organised national system	☐	☐	☐

If you wish, you can explain:
……
……

50. What are your reservations about the cervical cancer screening test?

**Tick one box per line**	**Yes**	**No**
I find the test painful	☐	☐
I am afraid of the result	☐	☐
I am afraid I will have to pay	☐	☐
I have other priorities	☐	☐

Other, specify: ……

C/ PERCEPTION OF PUBLIC HEALTH PROGRAMMES

51. Regarding organised **breast cancer screening,** how would you rate the items in the table below?

Tick one box per line	Important	Little or not important
Ensure that access to mammography facilities is subject to equal standards throughout the territory	☐	☐
Benefit from a reading of the mammogram by two radiologists	☐	☐
Benefit from 100% coverage of mammography without advance payment of costs	☐	☐
Have access to quality information	☐	☐
Be informed about the benefits and risks of screening	☐	☐
Be invited every two years by letter	☐	☐

Other, specify: ……………………………………………………………………………………………………….......

52. Regarding organised **colorectal cancer screening,** how would you rate the items in the table below?

Tick one box per line	Important	Little or not important
Being able to carry out the test at home	☐	☐
Benefit from a quality analysis of the test	☐	☐
Benefit from 100% coverage of mammography without advance payment of costs	☐	☐
Have access to quality information	☐	☐
Be informed about the benefits and risks of screening	☐	☐
Be invited every two years by letter	☐	☐

Other, specify: ……

53. What would you say about cancer screening programmes?

Tick one box per line	Yes	No	No opinion
They are legitimate in terms of public health	☐	☐	☐
They go against the freedom of the individual	☐	☐	☐

If you wish, you can explain: …………………………………………………………………………..

……

Name of the regional coordination cancer screening centre

Code category

54. What do you think of cancer screening campaigns (television, radio, *etc.*)?

Tick one box per line	Yes	No	No opinion
They allow you to be well informed	☐	☐	☐
They are indispensable	☐	☐	☐
They are well designed	☐	☐	☐
They generate anxiety	☐	☐	☐
There are too many of them	☐	☐	☐
They are unbearable	☐	☐	☐

If you wish, you can explain:

……

55. Detecting cancers at an early stage reduces the costs of cancer care and treatment for the community. In your opinion, is this an argument for participating in screening?

☐ Yes ☐ No ☐ No opinion

If you wish, you can explain:

……

……

56. Do you have confidence in public health vaccination programmes?

☐ Yes ☐ No ☐ No opinion

If you wish, you can explain:

……

……

57. Does participating in a public health programme seem to you to be a civic-minded attitude?

☐ Yes ☐ No ☐ No opinion

If you wish, you can explain:

……

58. You can leave your general comments on this survey here:

……

……

Thank you for your participation

All you have to do is put the questionnaire in the envelope you received. No need for a stamp, you can put it directly into the letterbox.

Name of the regional coordination cancer screening centre

Code category
